# Variance component analysis to assess protein quantification in biomarker validation: application to selected reaction monitoring-mass spectrometry

**DOI:** 10.1186/s12859-018-2075-8

**Published:** 2018-03-01

**Authors:** Amna Klich, Catherine Mercier, Laurent Gerfault, Pierre Grangeat, Corinne Beaulieu, Elodie Degout-Charmette, Tanguy Fortin, Pierre Mahé, Jean-François Giovannelli, Jean-Philippe Charrier, Audrey Giremus, Delphine Maucort-Boulch, Pascal Roy

**Affiliations:** 1 Service de Biostatistique-Bioinformatique, Hospices Civils de Lyon, 162, avenue Lacassagne, F-69003 Lyon, France; 2 Université de Lyon, Lyon, France; 3 PRABI, Université Lyon 1, Villeurbanne, France; 4 CNRS UMR 5558, LBBE, Équipe Biostatistique Santé, Villeurbanne, France; 5 Université Grenoble-Alpes, F-38000 Grenoble, France; 6 Commissariat à l’Énergie Atomique, Laboratoire d’Électronique et de Technologie de l’Information, MINATEC Campus, Département Micro-technologies pour la Biologie et la Santé, F-38054 Grenoble, France; 7 Innovation Unit, Technology Research Department, bioMérieux, F-69280 Marcy l’Étoile, France; 8 Innovation Unit, Technology Research Department, bioMérieux, F-38000 Grenoble, France; 9 Intégration du Matériau au Système (Université de Bordeaux, CNRS, Bordeaux Aquitaine INP), F-33400 Talence, France; 10 Present Address: Villeurbanne, France

**Keywords:** Mass spectrometry, SRM, Validation biomarkers, Technical variability, Experimental design, Variance component analysis

## Abstract

**Background:**

In the field of biomarker validation with mass spectrometry, controlling the technical variability is a critical issue. In selected reaction monitoring (SRM) measurements, this issue provides the opportunity of using variance component analysis to distinguish various sources of variability. However, in case of unbalanced data (unequal number of observations in all factor combinations), the classical methods cannot correctly estimate the various sources of variability, particularly in presence of interaction. The present paper proposes an extension of the variance component analysis to estimate the various components of the variance, including an interaction component in case of unbalanced data.

**Results:**

We applied an experimental design that uses a serial dilution to generate known relative protein concentrations and estimated these concentrations by two processing algorithms, a classical and a more recent one. The extended method allowed estimating the variances explained by the dilution and the technical process by each algorithm in an experiment with 9 proteins: L-FABP, 14.3.3 sigma, Calgi, Def.A6, Villin, Calmo, I-FABP, Peroxi-5, and S100A14. Whereas, the recent algorithm gave a higher dilution variance and a lower technical variance than the classical one in two proteins with three peptides (L-FABP and Villin), there were no significant difference between the two algorithms on all proteins.

**Conclusions:**

The extension of the variance component analysis was able to estimate correctly the variance components of protein concentration measurement in case of unbalanced design.

**Electronic supplementary material:**

The online version of this article (10.1186/s12859-018-2075-8) contains supplementary material, which is available to authorized users.

## Background

In the recent years, there has been a growing interest in using high throughput technologies to discover biomarkers. Because of the random sampling of the proteome within populations and the high false discovery rates, it became necessary to validate candidate biomarkers through quantitative assays [1]. ELISAs (Enzyme-Linked Immunosorbent Assays) have high specificities (because they often use two antibodies against the candidate biomarker) and high sensitivities that allow quantifying some biomarkers in human plasma. However, the limits with ELISA are the restricted possibility of performing multiple assays, the unavailability of antibodies for every new candidate biomarker, and the long and expensive developments of new assays [2]. The absolute quantification of protein biomarkers by mass spectrometry (MS) has naturally emerged as an alternative [3]. Eckel-Passow et al. [4] have discussed the difficulties of achieving good repeatability and reproducibility in MS and expressed the need for more research dedicated to proteomics data, including signal processing, experimental design, and statistical analysis.

In selected reaction monitoring (SRM, a specific form of multiple reaction monitoring, MRM) [5], the issues are somewhat different and offer the opportunity to use variance component analysis to investigate repeatability, reproducibility, and other sources of variability [6]. However, when the data are unbalanced (unequal number of observations in all possible factor combinations), classical methods cannot estimate correctly the various sources of variability, particularly in presence of interaction.

The present paper proposes an extension of the variance component analysis via the adjusted sum of squares that estimates correctly the various sources of variability of protein concentration on unbalanced data. This analysis is applied with an experimental design that uses a serial dilution to generate known relative protein concentrations and allows for a few sources of variation. Two processing algorithms, a classical and a more recent one (namely, NLP and BHI, respectively) are used to estimate protein concentration.

This analysis allowed an initial investigation of the performance of the new algorithm and a first comparison with the classical algorithm. In addition, the results given by the two algorithms are compared with those obtained by ELISA.

## Methods

### Sample preparation

Because the true proteomic profiles in biological samples are unknown, an artificial “biological variability” (herein called “dilution variability”) was generated by serial dilution (an experiment close to the design of Study III by Addona et al. [7]). Twenty-one target proteins (bioMérieux, Marcy l’Étoile, France) were considered: 14.3.3 sigma, binding immunoglobin protein (BIP), Calgizzarin or S100 A11 (Calgi), Calmodulin (Calmo), Calreticulin (Calret), Peptidyl-prolyl cis-trans isomerase A (Cyclo-A), Defensin α5 (Def-A5), Defensin α6 (Def.A6), Heat shock cognate 71 kDa protein (HSP71), Intestinal-Fatty Acid Binding Protein (I-FABP), Liver-Fatty Acid Binding Protein (L-FABP), Stress-70 protein mitochondrial (Mortalin), Protein Disulfide-Isomerase (PDI), Protein disulfide-isomerase A6 (PDIA6), Phosphoglycerate kinase 1 (PGK1), Retinol-binding protein 4 (PRBP), Peroxiredoxin-5 (Peroxi-5), S100 calcium-binding protein A14 (S100A14), Triosephosphate isomerase (TPI), Villin-1 (Villin), and Vimentin. These proteins were diluted in a pool of human sera (Établissement Français du Sang, Lyon, France). In other words, a parent solution (mixture of target proteins spiked in the pool of human sera) was used at dilutions 1, 1/2, 1/4, 1/8, and 1/16. This led to the use of six “samples” (serum pool + 5 dilutions). Five aliquots of 250 μL were taken from each sample: four for SRM and one for ELISA. In addition, eight extra aliquots of 250 μL of dilution 1/4 were used to estimate the digestion yield.

### Experimental design

**Fig. 1:**
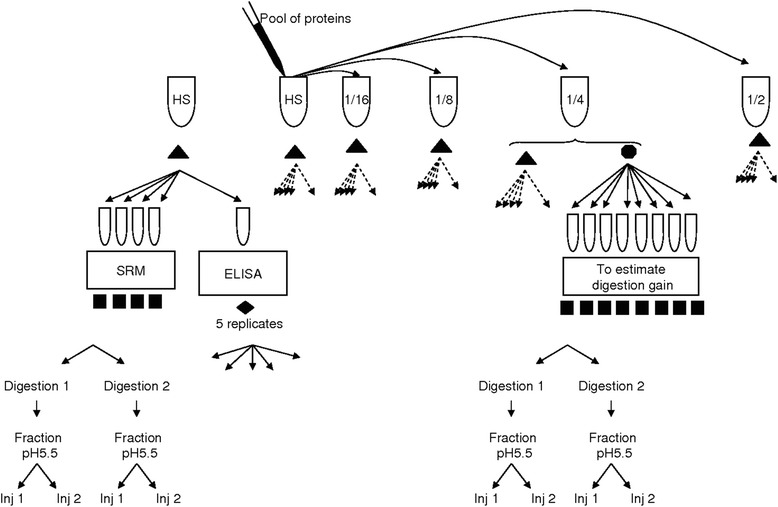
HS: pool of human serum - SRM: selected reaction monitoring - Inj: injection - The triangles indicate the samples destined for SRM and ELISA - The circle indicates the samples destined solely for estimating the digestion yield by SRM - The squares indicate the samples destined for reading by SRM readings - The diamond indicates the samples destined for ELISA readings

The experimental design is shown in Fig. 1. From each aliquot, two vials of 125 μL were taken for separate digestions. Labelled AQUA internal standards were added immediately before SRM-MS analysis then two injections (readings) were performed on each vial. SRM readings of the 24 aliquots (6 samples × 2 digestions × 2 injections) were carried out over 4 couples of days. Each set of samples had to be “read” over a couple of days because of equipment-related constraints (SRM does not allow analyzing all the samples in a single day). To avoid unexpected or uncontrolled biases, sample reading was made at random and two chromatographic columns were alternately used (for more details on SRM, see Additional file 1).

In the methodology associated with BHI algorithm, we used quality control (QC) measurements made daily before peptide reading to estimate the digestion yield. In parallel, each “extra” aliquot of dilution 1/4 was used on one reading day as QC measurement. From each “extra” aliquot, two vials of 125 μL were taken for digestion. The two vials were passed one at the start and the other at the end of the day then each vial was injected two times leading to the calibration of four digestion yields per day. The estimation of protein concentration with BHI algorithm on a given day changes according to the digestion yield estimated the same day.

With the classical NLP algorithm, the number of readings per sample was 16 (4 aliquots × 2 digestions × 2 injections). With the BHI algorithm, the number of readings per sample was 64 (4 aliquots × 2 digestions × 2 injections × 4 digestion yields).

For ELISA measurement of Liver-Fatty Acid Binding Protein (L-FABP), each sample provided five replicates. Each replicate was read four times leading to 20 readings per sample. Sample readings were made at random.

### Protein quantification methods

#### The BHI algorithm

**Fig. 2:**
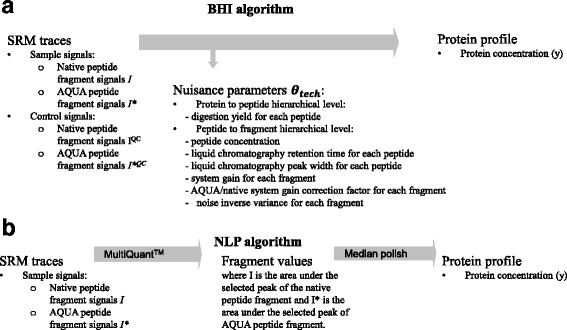
SRM: selected reaction monitoring- BHI algorithm: Bayesian Hierarchical algorithm- NLP algorithm: the classical algorithm- AQUA : Absolute QUAntification (labelled internal standard) - QC: quality control- θ_tech_ the set of latent technical parameters

The Bayesian Hierarchical Algorithm (BHI) is based on a full graphical hierarchical model of the SRM acquisition chain which combines biological and technical parameters (Fig. 2a, Tables 1 and 2).

**Table 1:** Parameters and variables involved in the SRM analytical chain model

Notation	Description	Range
t	Time	
*t*_*iln*_	Discrete time sample *n* for peptide *i* and fragment *l*. In experimental conditions where only one ion by peptide is followed, this element is labeled only by peptide identifier *i*	*i* = 1, …, *S* *l* = 1, …, *L* *n* = 1, …, *N*
*d*_*ip*_	Digestion factor defined by the number of peptides *i* present in protein *p*	*i* = 1, …, *S* *p* = 1, …, *P*
*g*_*ip*_	Digestion yield defined by the correction factor to apply to the digestion factor *d*_*ip*_to obtain ratio peptide/protein concentration	*i* = 1, …, *I* *p* = 1, …, *P*
*ξ*_*il*_	Peptide to fragment gain	*i* = 1, …, *S* *l* = 1, …, *L*
${\phi}_{il}^{\ast }$	Peptide to fragment gain correction factor for AQUA peptide	*i* = 1, …, *S* *l* = 1, …, *L*
*C*_*i*_(*τ*_*i*_, *λ*_*i*_)	Normalized chromatography peak response of peptide *i*	*i* = 1, …, *S*
*τ*_*i*_	Chromatography peak position	*i* = 1, …, *S*
*λ*_*i*_	Chromatography peak width	*i* = 1, …, *S*
*I*_*ikl*_(*t*_*il*_)	Transition signal at time *t*_*il*_	*i* = 1, …, *S* *k* = 1, …, *K* *l* = 1, …, *L*
*y*_*p*_	Protein *p* concentration	*p* = 1, …, *P*
*κ*_*i*_	Peptide *i* concentration before chromatography	*i* = 1, …, *S*
*ϱ*_*ik*_	Concentration of selected ion *k*of peptide *i* (precursor ion ^*k*^ of transition ^*l*^)	*k* = 1, …, *K*
*ϑ*_*kl*_	Concentration of selected fragment *l*of selected ion *k* (fragment of precursor ion *k* of transition *l*)	*l* = 1, …, *L*

**Table 2:** Hierarchical model equations of the SRM analytical chain for the native transition signals I and labeled transition signals I*

Quantity	Targeted protein	AQUA peptide standard	
Protein concentration	*y*_*p*_	No labeled protein introduced	*p* = 1, …, *P*
Peptide concentration before chromatography	$$ {\displaystyle \begin{array}{c}{H}_i(y)=\sum \limits_{p=1}^P{g}_{ip}{d}_{ip}{y}_p\\ {}{\kappa}_i={H}_i(y)+N\left({\gamma}_{\kappa}\right)\end{array}} $$	${\kappa}_i^{\ast }$	*i* = 1, …, *S*
Selected ion concentration before fragmentation	*ξ*_*i*_ *κ*_*i*_	${\xi}_i{\kappa}_i^{\ast }$	*i* = 1, …, *S*
Signal of transition at time *t*_*n*_	${\kappa}_i{\xi}_{il}{C}_{il}^T\left({\tau}_i,{\lambda}_i\right)\left({t}_n\right)$	${\kappa}_i^{\ast }{\xi}_{il}{\phi}_{il}^{\ast }{C}_{il}^T\left({\tau}_i,{\lambda}_i\right)\left({t}_n\right)$	*i* = 1, …, *S* *l* = 1, …, *L* *n* = 1, …, *N*
Resulting signals of selected children of targeted peptide^a^	$$ {\displaystyle \begin{array}{c}{G}_l\left(\boldsymbol{\kappa}, \boldsymbol{\xi}, \boldsymbol{\tau}, \boldsymbol{\lambda} \right)=\sum \limits_{i=1}^S\sum \limits_{l=1}^L{\kappa}_i{\xi}_{il}{C}_{il}^T\left({\tau}_i,{\lambda}_i\right)\\ {}{I}_l={G}_l\left(\boldsymbol{\kappa}, \boldsymbol{\xi}, \boldsymbol{\tau} \right)+N\left({\gamma}_{nl}\right)\end{array}} $$	$$ {\displaystyle \begin{array}{c}{G}_l^{\ast}\left({\boldsymbol{\kappa}}^{\ast},\boldsymbol{\xi}, {\boldsymbol{\phi}}^{\ast},\boldsymbol{\tau}, \boldsymbol{\lambda} \right)=\sum \limits_{i=1}^S\sum \limits_{l=1}^L{\kappa}_i^{\ast }{\xi}_{il}{\phi}_{il}^{\ast }{C}_{il}^T\left({\tau}_i,{\lambda}_i\right)\\ {}{I}_l^{\ast }={G}_l^{\ast}\left({\boldsymbol{\kappa}}^{\ast},\boldsymbol{\xi}, {\boldsymbol{\phi}}^{\ast},\boldsymbol{\tau} \right)+N\left({\gamma}_{nl}^{\ast}\right)\end{array}} $$	*i* = 1, …, *S* *l* = 1, …, *L*

^a^Bold notation stands for vectors

To estimate all these parameters, two calibrations are required: the use of quality control (QC) samples measured each day for calibration (at protein level) and the use of AQUA peptides for calibration (at peptide level) (see section “Experimental design”). This set of measurements leads to a set of equations that captures the links between the unknown latent variables and parameters to estimate and the known SRM measurement. Estimating a protein concentration requires estimating, at the same time, the technical parameters included in the model.

Table 1 shows all the parameters and variables involved in the description of the SRM analytical chain model. Let ***θ***_***tech***_ be the set of latent technical parameters that describes the SRM acquisition chain:

$$ {\boldsymbol{\theta}}_{\boldsymbol{tech}}=\left(\boldsymbol{\kappa}, \boldsymbol{\tau}, \boldsymbol{\lambda}, \boldsymbol{\xi}, {\boldsymbol{\phi}}^{\ast },\boldsymbol{\gamma}, {\boldsymbol{\gamma}}^{\ast}\right) $$

Table 2 shows the hierarchical model that links protein concentration ***y*** to the native transition signals ***I*** of native peptides and the labeled transition signals ***I***^***∗***^ of AQUA peptides.

The BHI algorithm has to solve the inverse problem and compute protein concentration ***y*** and technical parameters ***θ***_***tech***_. This problem is solved in a Bayesian framework [8–15]. Table 3 shows the distribution type used for each variable in this Bayesian framework.

**Table 3:** Distribution type for each variable of the SRM acquisition chain

Hierarchical level	Variable	Analytic expression distribution^a^	Distribution type
Transition	Noise	$$ {\displaystyle \begin{array}{c}p\left(\boldsymbol{I}|\boldsymbol{\kappa}, \boldsymbol{\xi}, \boldsymbol{\tau}, \boldsymbol{\lambda}, \gamma \right)\sim \prod \limits_{l=1}^L\mathit{\exp}\left(-\frac{1}{2}{\gamma}_n{\left\Vert {I}_l-{G}_l\left(\kappa, \xi, \tau, \lambda \right)\right\Vert}^2\right)\\ {}p\left({I}^{\ast }|\boldsymbol{\kappa}, \boldsymbol{\xi}, {\boldsymbol{\phi}}^{\ast},\boldsymbol{\tau}, \boldsymbol{\lambda}, {\gamma}^{\ast}\right)\sim \prod \limits_{l=1}^L\mathit{\exp}\left(-\frac{1}{2}{\gamma}_n^{\ast }{\left\Vert {I}_l^{\ast }-{G}_l^{\ast}\left(\kappa, \xi, {\phi}^{\ast },\tau, \lambda \right)\right\Vert}^2\right)\end{array}} $$	Normal
Peptide	Peptide to fragment gain	$p\left(\boldsymbol{\xi} \right)\sim \prod \limits_{i=1}^S\mathit{\exp}\left(-\frac{1}{2}{\gamma}_{\xi}^i{\left({\xi}_i-{m}_{\xi_i}\right)}^2\right)$	Normal
	Peptide to fragment gain correction factor	$p\left({\boldsymbol{\phi}}^{\ast}\right)\sim \prod \limits_{i=1}^S\mathit{\exp}\left(-\frac{1}{2}{\gamma}_{\phi}^{\ast }{\left({\phi}_i^{\ast }-1\right)}^2\right)$	Normal
	Noise inverse variance	$$ {\displaystyle \begin{array}{c}p\left({\gamma}_n\right)\sim \frac{\gamma_n^{\alpha_n-1}}{\beta_n^{\alpha_n}\Gamma \left({\alpha}_n\right)}\mathit{\exp}\left(-\frac{\gamma_n}{\beta_n}\right)\\ {}p\left({\gamma}_n^{\ast}\right)\sim \frac{\gamma_n^{\ast \left({\alpha}_n-1\right)}}{\beta_n^{\alpha_n}\Gamma \left({\alpha}_n\right)}\mathit{\exp}\left(-\frac{\gamma_n^{\ast }}{\beta_n}\right)\end{array}} $$	Gamma
	Peak retention time	$p\left(\boldsymbol{\tau} \right)\sim \prod \limits_{i=1}^SU\left({\tau}_i;{\tau}_i^m,{\tau}_i^M\ \right)$	Uniform
	Peak width	$p\left(\boldsymbol{\lambda} \right)\sim \prod \limits_{i=1}^SU\left({\lambda}_i;{\lambda}_i^m,{\lambda}_i^M\ \right)$	Uniform
	Peptide concentration	$p\left(\boldsymbol{\kappa} |\boldsymbol{y}\right)\sim \prod \limits_{i=1}^S\mathit{\exp}\left(-\frac{1}{2}{\gamma}_{\kappa }{\left({\kappa}_i-{H}_i(y)\right)}^2\right)$	Normal
Protein	Protein concentration	$p\left(\boldsymbol{y}\right)\sim \prod \limits_{p=1}^P\mathit{\exp}\left(-\frac{1}{2}{\gamma}_x^p{\left({y}_p-{m}_{y_p}\right)}^2\right)$	Normal
	Digestion yield	$p\left(\boldsymbol{g}\right)\sim \prod \limits_{i=1}^S\prod \limits_{p=1}^P\mathit{\exp}\left(-\frac{1}{2}{\gamma}_g{\left({g}_{ip}-{m}_g\right)}^2\right)$	Normal

^a^Bold notation stands for vectors

To estimate together the protein concentration and the parameters, we used the native transition signals **I** with the labeled transitions signals I*. Regarding the labeled signal, the peptide concentration is known but the transition gains and the inverse variance of the noise in the AQUA signal have to be estimated.

Using the distributions defined in Table 3, the full a posteriori distribution *p*(***y***, ***θ***_***tech***_| ***I***, ***I***^***∗***^) can be approximated as follows:

$$ p\left(\boldsymbol{y},{\boldsymbol{\theta}}_{\boldsymbol{tech}}|\boldsymbol{I},{\boldsymbol{I}}^{\ast}\right)\sim p\left(\boldsymbol{y}\right)p\left(\boldsymbol{\kappa} |\boldsymbol{y}\right)p\left(\boldsymbol{\tau} \right)p\left(\boldsymbol{\lambda} \right)p\left(\boldsymbol{\xi} \right)p\left({\boldsymbol{\phi}}^{\ast}\right)p\left(\boldsymbol{\gamma} \right)p\left({\boldsymbol{\gamma}}^{\ast}\right)p\left(\boldsymbol{I}|\boldsymbol{\kappa}, \boldsymbol{\xi}, \boldsymbol{\tau}, \boldsymbol{\lambda}, \boldsymbol{\gamma} \right)p\left({\boldsymbol{I}}^{\ast }|\boldsymbol{\kappa}, \boldsymbol{\xi}, {\boldsymbol{\phi}}^{\ast },\boldsymbol{\tau}, \boldsymbol{\lambda}, {\boldsymbol{\gamma}}^{\ast}\right) $$

The protein concentration and the parameters are estimated by the expectation of this a posteriori (EAP) distribution. This EAP is defined as follows:

$$ \left(\overset{\sim }{\boldsymbol{y},}\overset{\sim }{{\boldsymbol{\theta}}_{\boldsymbol{tech}}}\right)= EAP\left(\left(\boldsymbol{y},{\boldsymbol{\theta}}_{\boldsymbol{tech}}\right)\right) $$

$$ EAP\left(\left(\boldsymbol{y},{\boldsymbol{\theta}}_{\boldsymbol{tech}}\right)\right)=\int \left(\boldsymbol{y},{\boldsymbol{\theta}}_{\boldsymbol{tech}}\right)p\left(\boldsymbol{y},{\boldsymbol{\theta}}_{\boldsymbol{tech}}|\boldsymbol{I},{\boldsymbol{I}}^{\ast}\right)d\boldsymbol{y}d{\boldsymbol{\theta}}_{\boldsymbol{tech}} $$

Computing the EAP is achieved with methods based on Markov Chain Monte-Carlo (MCMC) procedure and hierarchical Gibbs structure. The algorithm performs sequentially a random sampling of each parameter (***y***, ***θ***_***tech***_) from the a posteriori distribution and conditionally on the previously sampled parameters, and iterates. The parameters are sampled in the following order: ***κ***, ***τ***, ***λ***, ***ξ***, ***ϕ***^***∗***^, ***γ***, ***γ***^***∗***^. In the case of a Normal distribution, the sampling is achieved knowing explicitly the mean and the inverse variance of the distribution. In the case of a uniform distribution, the sampling is achieved using one iteration of a Metropolis-Hastings random walk. After a fixed number of iterations, the algorithm computes the empirical mean of each parameter after a warm-up index. This index defines the number of iterations at convergence towards the a posteriori distribution.

Here, we supposed that the digestion yields are known. With BHI, we have introduced a protocol for estimating the digestion yields. We used the control signals measured on the quality-control sample. We assumed that the digestion factors *d*_*ip*_ defined by the number of peptides *i* present in protein p are known. The digestion yield *g*_*ip*_ is defined by the correction factor to apply to the digestion factor to obtain ratio peptide/protein concentration. Note here that a matrix formulation allows handling non-proteotypic peptides shared by several proteins. The Control signals combine both native transition signals ***I***^***QC***^ and labeled transition signals ***I***^***∗QC***^. According to the above-described Bayesian algorithm, the unknown becomes the digestion yield of each peptide instead of the protein concentration. Here too, estimating the EAP calls for a MCMC algorithm with hierarchical Gibbs structure. This calibration is done once for each calibration batch selecting one quality control measurement. This process may be generalized to the cases where several quality control measurements are available by combining within the EAP computation the information delivered by each measurement.

The BHI algorithm includes an automated selection to initialize the peak position that is based on the set of transitions associated with each peptide. It computes the product of the traces and searches for the position of the maximum value on this product. This way, only the peaks present in all traces are detected.

Algorithm BHI involves a fusion of the information delivered by all traces. This improves the algorithm robustness when the number of traces is large. In fact, generally, processing algorithms for protein quantification are most performant with proteins of ≥3 peptides and peptides with ≥3 transitions [16].

#### The NLP algorithm

The NLP algorithm (Fig. 2b) is based on the median value, over all transitions, of the log-transformation of ratio native transition peak area/labeled transition peak area. This algorithm is derived from a gold standard algorithm used for oligonucleotide array analysis [17]. The peaks are detected by MultiQuant™ software (AB Sciex, France). These peaks are checked by an operator who decides whether a signal of the labeled internal peptide standard AQUA does not make sense and should be considered as missing or whether a too low or absent signal of the native transition should be assigned value 0.

The NLP algorithm uses, as input data, a normalized and log-transformed quantity *t* defined by: $t= Ln\left(1+\frac{I}{I^{\ast }}\right)$ where I represents the area under the peak of a given native transition and I* the area under the peak of the labeled transition.

#### Elisa

Only protein L-FABP was concerned. The concentration of this protein was measured using Vidas HBSAg® protocol, the 2C9G6/5A8H2 antibody pair, and Vidas® analyser (bioMérieux, Marcy-l’Étoile, France).

### Statistical modeling and analysis

In this article, the performance of each algorithm in SRM and the performance of ELISA were defined as the ability to find the concentrations generated by serial dilution. This ability was estimated by the linear slope and the variance decomposition of the linear model that links the measured to the theoretical protein concentration generated by dilution. The best performance corresponds to the highest part of dilution variance (explained by the dilution) and the lowest part of technical variance (explained by the measurement error and lab procedures).

Only proteins that have a correlation coefficient ≥ 0.7 between theoretical and measured concentration with either NLP or BHI algorithm were selected for the statistical analysis.

### Linearity analysis

For each algorithm and each protein, a linear regression model was built to link the protein concentration *y* with the theoretical protein concentration *x*. A log2 transformation of the measurements was applied to stabilize the variance. Because of the two-fold dilution, the log2 transformation was applied to *x* and *y*. With this transformation, the regression line is expected to have a slope close to 1.

Because the reading on a given couple of days may influence the relationship between the measured and the theoretical concentration of each protein, the model included a slope and an intercept for each day-couple; this comes to include an interaction term between protein concentration and day-couple. A fixed effects model was applied and ‘sum to zero contrasts’ were used to obtain estimations of the mean intercept and the mean slope as follows:

$$ {y}_{ijr}={\beta}_0+{\beta}_{0j}{D}_j+{\beta}_1{x}_{ijr}+{\beta}_{1j}{x_{ijr}}^{\ast }{D}_j+{\varepsilon}_{ijr}\ \left(\mathrm{Model}\kern0.5em 1\mathrm{S}\right) $$

*i*, *j*, and *r* correspond respectively to the sample, the day-couple, and the digestion-injection step. Parameters *β*_0_ and *β*_1_ are respectively the mean intercept and the mean slope of the regression line between the log2 values of the measured protein concentrations and the log2 values of the theoretical protein concentrations, *β*_0*j*_and *β*_1*j*_ being, respectively, the two-day-reading effects on the mean intercept and the mean slope. D is for a day-couple.

In parallel, a log2 transformation was applied to ELISA measurements too. These measurements were then analyzed by a linear model (Model 1E) that included the theoretical concentration *x*, the reading order T, and the interaction between them:

$$ {y}_{ijr}={\beta}_0+{\beta}_1{x}_{ijr}+{\beta}_{0j}{T}_j+{\beta}_{1j}{x_{ijr}}^{\ast }{T}_j+{\varepsilon}_{ijr}\ \left(\mathrm{Model}\kern0.5em 1\mathrm{E}\right) $$

*i*, *j*, and *r* correspond, respectively, to the sample, the reading order, and the replicate.

### Variance decomposition

In this work, the data processed by the NLP algorithm included null intensities and missing values (see Additional file 2). These values were excluded after log2 transformation. As their number was unequal between the couple of day readings, the data were considered unbalanced.

To quantify the components of the variance, we calculated adjusted sums of squares by comparing complete Model 1S with each of its nested models. The nested models are shown below: Model 2S included only the effect of the theoretical concentration, Model 3S only the effect of the two-day measurement, and 4S both effects without interaction between them:

$$ {y}_{ijr}={\beta}_0+{\beta}_1{x}_{ijr}+{\varepsilon}_{ijr}\ \left(\mathrm{Model}\ 2\mathrm{S}\right) $$

$$ {y}_{ijr}={\beta}_0+{\beta}_{0j}{D}_j+{\varepsilon}_{ijr}\ \left(\mathrm{Model}\ 3\mathrm{S}\right) $$

$$ {y}_{ijr}={\beta}_0+{\beta}_{0j}{D}_j+{\beta}_1{x}_{ijr}+{\varepsilon}_{ijr}\ \left(\mathrm{Model}\ 4\mathrm{S}\right) $$

**Fig. 3:**
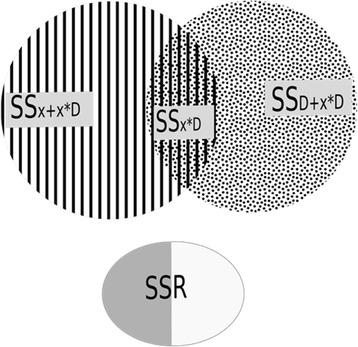
Venn Diagrams showing the variance components

Table 4 and Fig. 3 present the components of the analysis of variance. The dilution variance and its interaction with the two-day measurement effect, was calculated as the difference between Model 3S and Model 1S residual sums of squares. The lab procedure variance corresponds to the variance explained by the two-day measurement effect and its interaction with the theoretical concentration was calculated as the difference between Model 2S and Model 1S residual sums of squares. The variance explained by the sole interaction between the theoretical concentration and the two-day measurement was calculated by the difference between Model 4S and Model 1S residual sums of squares.

**Table 4:** Variance decomposition of Model 1S

Source of variation	DF	Adjusted sum of squares
Theoretical concentration and interaction	J	*SS*(*x* + *x*^∗^*D*|*D*)=*RSS*(Model 3S) − *RSS*(Model 1S)
Two-day measurement and interaction	2(J-1)	*SS*(*D* + *x*^∗^*D*|*x*)=*RSS*(Model 2*S*) − *RSS*(Model 1*S*)
Interaction	(J-1)	*SS*(*x*^∗^*D*|*x*, *D*)=*RSS*(Model 4*S*) − *RSS*(Model 1*S*)
Residual variation	IJR-2 J	$RSS\left(\mathrm{Model}\ 1S\right)=\sum \limits_{ijr}{\left({y}_{ijr}-{\widehat{y}}_{ijr}\right)}^2$
Measurement error	(R-1)*I*J	$\sum \limits_{ij r}{\left({y}_{ij r}-{\overline{y}}_{ij\bullet}\right)}^2$
Lack of fit	IJ-2 J	$\sum \limits_{ij r}{\left({\widehat{y}}_{ij r}-{\overline{y}}_{ij\bullet}\right)}^2$

*DF* degrees of freedom, *I* number of samples, *J* number of couples of days, *R* number of digestion-injections -${\overline{y}}_{ij\bullet }$: mean of digestion-injection replicate measurements of each sample and each couple of days - ${\widehat{y}}_{ijr}$: predicted measurements - RSS: residual sum of squares - SS: sum of squares

The residual variance was split into two components [18]: 1) the measurement error due to instrumental and algorithmic errors, which was calculated as the sum of the squares of the differences between the injection replicate values and their mean, and 2) the lack of fit of the model.

For ELISA, the same analysis of variance was applied to Model 1E. Each component of the sum of squares was divided by the total sum of squares and expressed as a percentage. This helped comparing the three methods (ELISA and the two processing algorithms for SRM).

Two Wilcoxon signed-rank tests were used on all proteins to test, first the difference between the parts of dilution variance then between the parts of technical variance given by the two processing algorithms. These two tests are not independent and correspond to a single test in case of absence of interaction.

## Results

Among all results obtained for all protein reads, the correlation coefficient between the theoretical concentration and the measured protein concentration was ≥0.7 in 9 out of 21 proteins: L-FABP, 14.3.3 sigma, Calgi, Def.A6, Villin, Calmo, I-FABP, Peroxi-5, and S100A14 (Additional files 3 and 4). The correlation coefficient was ≥0.7 with BHI and NLP in proteins 14.3.3 sigma, Calgi, Def.A6, and Villin. This coefficient was ≥0.7 with NLP only in Calmo, I-FABP, Peroxi-5, and S100A14 and with BHI only in L-FABP.

### Linearity analysis

Table 5 and Additional file 5 summarize the analysis of variance in each linear model relative to each of the 9 above-cited proteins. Table 5 shows also the mean slopes of these linear models.

**Table 5:** Estimations of the mean slope and results of variance decomposition

Protein and algorithm	Peptide number	Mean slope	Theoretical concentration + interaction^a^	Interaction	Two-day process+ interaction^b^	Measurement error^b^	Total^b^	Lack of fit
L-FABP	3							
NLP		0.64	27.2	14.4	24.9	45.7	70.6	16.9
BHI		0.72	54.8	3.9	5.7	30.5	36.2	12.9
ELISA^c^		0.84	98.1	0.1	0.6	0.3	0.8	1.1
Villin	3							
NLP		1.14	51.1	5.6	9.9	14.4	24.3	28.8
BHI		0.98	74.7	1.7	1.8	16.8	18.6	8.3
14.3.3 sigma	1							
NLP		1.09	69.8	5.8	16.9	16.4	33.3	9.5
BHI		0.77	87.2	0.7	2.1	10.1	12.2	1.4
Calgi	2							
NLP		1.02	86.2	1.8	6	6.7	12.7	2.9
BHI		0.81	93.5	0.1	1	4.3	5.3	1.3
Def.A6	1							
NLP		0.97	97.6	0.1	0.1	2.	2.1	0.2
BHI		0.95	97.3	0.0	0.3	2.2	2.5	0.2
Calmo	1							
NLP		0.55	87.1	0.6	8	4.7	12.7	2.4
BHI		0.32	19.8	0.8	16	52.	68.1	12.9
I-FABP	1							
NLP		0.86	89.3	0.4	2	5.1	7.1	2.5
BHI		0.22	2.8	2.1	5.9	86.	91.9	7.4
Peroxi-5	1							
NLP		0.69	80.4	1.5	2.5	15.2	17.6	2.2
BHI		0.30	27.3	15.2	24	51.5	75.5	12.4
S100A14	2							
NLP		0.88	85.9	15.9	21.4	4.5	25.9	7.8
BHI		0.34	30.2	20.3	41.4	34.6	76	14.1

The results of variance decomposition (columns 4 to 9) are expressed as percentages, ^a^ Reflects the dilution variance. ^b^ Reflects the technical variance. ^c^ Results stemming from the reading order (not the two-day readings)

For L-FABP and Villin, the BHI algorithm gave a higher dilution variance and a lower technical variance than the NLP algorithm. In addition, the mean slope of Model 1S was closer to 1 with the BHI algorithm than with the NLP algorithm.

The BHI algorithm gave various results with the other proteins that have less than three peptides. For 14.3.3 sigma and Calgi, the BHI algorithm gave a higher dilution variance and a lower technical variance than the NLP algorithm. Def.A6 gave similar results with both algorithms. The BHI algorithm gave lower dilution variance and higher technical variance than the NLP algorithm with Calmo, I-FABP, Peroxi-5, and S100A14. Moreover, 14.3.3 sigma, and Calgi, Calmo, I-FABP, Peroxi-5, and S100A14, the mean slope of Model 1S was closer to 1 with the NLP than with the BHI algorithm.

But, on all proteins, the dilution variances and the technical variances were not significantly different between the two algorithms (*p*-values = 0.35 in both comparisons with Wilcoxon signed ranks test).

With L-FABP, ELISA gave higher dilution variance and lower technical variance than SRM with the two algorithms in terms of dilution variance and technical variance. Besides, the mean slope of Model 1E was closer to 1 than the mean slopes obtained with Model 1S with either the BHI or the NLP algorithm.

### Technical variance components

The part of the measurement error was the highest part of the technical variance with the BHI with L-FABP, 14.3.3 sigma, Calgi, Def.A6, and Villin and with the NLP with Calmo, I-FABP, Peroxi-5, and S100A14. The other components of the technical variance (i.e., the two-day measurement and the interaction between this measurement and the theoretical concentration) included a variability of the intercept and a variability of the slope of the regression lines relative to the 4 day-couples.

**Fig. 4:**
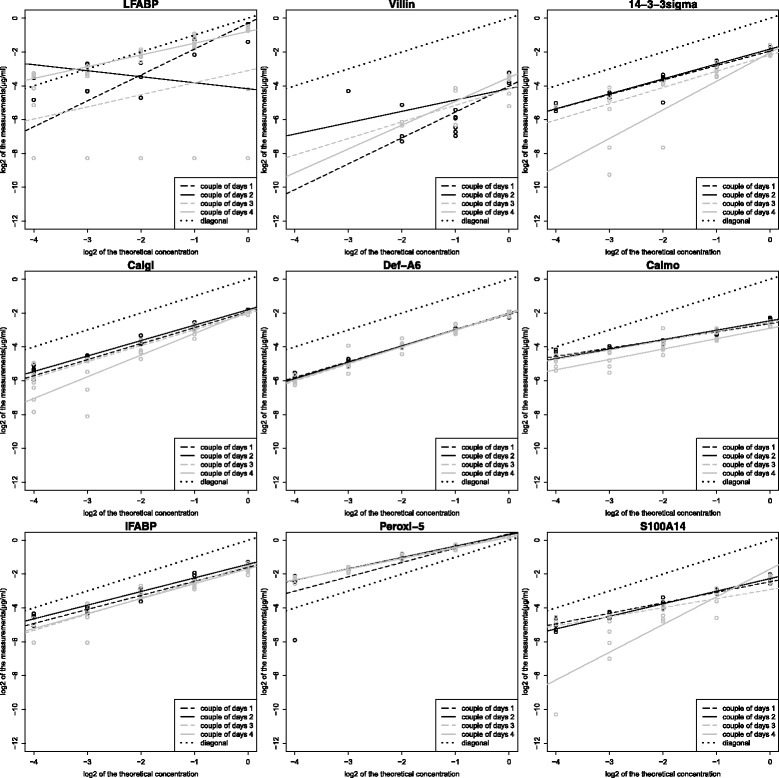
Two-day reproducibility of the linear model slopes with the NLP algorithm on the log2-log2 scale. In each panel, the solid line represents the diagonal regression line

**Fig. 5:**
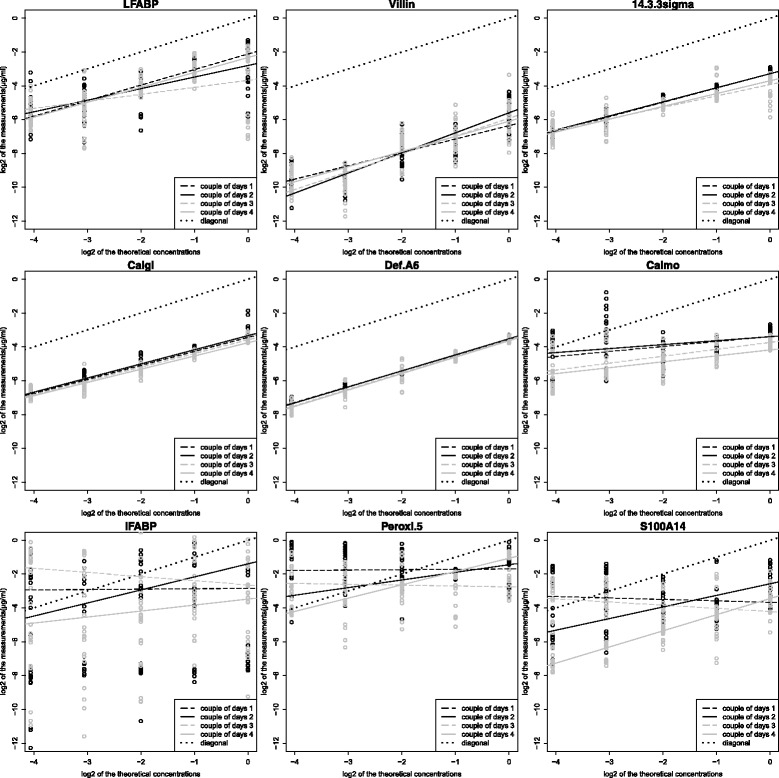
Two-day reproducibility of the linear model slopes with the BHI algorithm on the log2-log2 scale. In each panel, the solid line represents the diagonal regression line

**Fig. 6:**
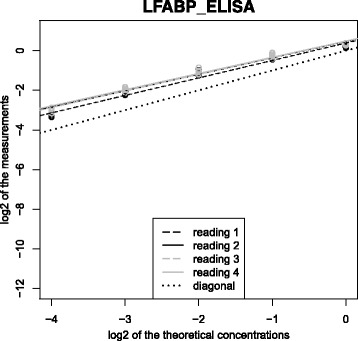
Reading-order reproducibility of the linear model slopes with L-FABP protein quantification by ELISA on the log2-log2 scale. In each panel, the solid line represents the diagonal regression line

Figures 4 and 5 show the relationships between the theoretical and the measured protein concentrations on the log2-log2 scale for the 9 proteins with algorithms NLP and BHI, respectively. Figure 6 shows the relationship between the theoretical and the measured L-FABP concentration with ELISA. In these figures, for L-FABP, 14.3.3 sigma, Calgi, Def.A6, and Villin, the four regression lines relative to the 4 day-couples were more grouped with BHI than with NLP. This means that the part of the variance due to the two-day measurement process and the interaction between this process and the theoretical concentration is smaller with BHI than with NLP (also shown in Table 5). For Def.A6, this part of the variance was very low with both algorithms; thus, the part due to the measurement error was the highest part of the technical variance.

In Figs. 4 and 5, for Calmo, I-FABP, Peroxi-5, and S100A14, the four regression lines relative to the 4 day-couples were more grouped with NLP than with BHI. This means that the part of the variance due to the two-day measurement process and the interaction between this process and the theoretical concentration is smaller with NLP than with BHI.

## Discussion

The present article proposes an extension of variance component analysis via adjusted sums of squares by estimating correctly the various sources of variability on unbalanced data. This analysis allows estimating separately the dilution variability and the technical variability. In an application to protein concentration estimation by two processing algorithms (NLP and BHI), this extension allowed algorithm performance quantification and comparison. The results showed that the performance of each algorithm as reflected by the dilution and the technical variance depended on the protein and that, on all proteins, there were no significant difference between the two algorithms.

Other statistical modeling frameworks were proposed for protein quantification in SRM experiments. SRMstats [19] uses a linear mixed-effects model to compare distinct groups (or specific subjects from these groups). Its primary output is a list of proteins that are differentially abundant across settings and the dependent variables are the log-intensities of the transitions. Here, a simple linear model was used to find the theoretical protein concentrations generated by serial dilution, the primary outputs are the components of the variance (essentially, the variance component explained by the serial dilution) and the dependent variable is the protein concentration estimated by the quantification algorithm on the basis of the ratio of native to labeled transitions (see parts “The BHI algorithm” and “The NLP algorithm”).

In the publication of Xia et al. [6], the reproducibility of the SRM experiment was assessed by decomposing the variance into parts attributable to different factors using mixed effects models. The sequential ANOVA was used to quantify the variance components of the fixed effects. However, when the data are unbalanced, the sequential ANOVA cannot correctly estimate the different parts of the variance: with balanced data, one factor can be held constant whereas the other is allowed to vary independently. This desirable property of orthogonality is usually lost with unbalanced data which generated correlations between factors. With such data, the use of adjusted sums of squares (Type II and Type III sum of squares in some statistical analysis programs) [20–23] is then an appropriate alternative to the sequential sums of squares. With Type II, each effect is adjusted on all other terms except their interactions; thus one limitation is that this approach is not applicable in the presence of interactions. With Type III, each effect is adjusted on all other terms including their interactions but one major criticism is that some nested models used for estimating the sums of squares are unrealistic [24] because these models that include interaction terms between effects do not allow for all effects (For example in a three-way Table (A, B and C), the model that includes interaction A*B*C does not include all effects A, B, C). Here, we propose an approach that responds to this criticism.

In SRM protein quantification, the BHI algorithm revealed a higher part of the whole dilution variance and a lower part of the whole technical variance vs. NLP with L-FABP and Villin, the only two proteins that have three peptides. This is one limitation of the present study because assessing the performance of the BHI algorithm requires other proteins with three peptides or proteins with more than three peptides.

In comparing the two algorithms using proteins with less than three peptides, the difference in measurement results may be explained by differences in the proprieties of these algorithms. Firstly, the NLP algorithm is supervised and assesses the quality of both the native and the labeled transition before estimating their ratio (spectrum visualization by an operator) whereas the BHI algorithm is automatic and gives directly an estimation of this ratio, including a weighting of each transition according to the estimated level of noise. When the signals of the labeled transition are not detectable, the measures do not make sense and are considered missing for the NLP algorithm but give incorrect values with the BHI algorithm. For Peroxi-5 and S100A14 proteins, 27% and 36% of the values of the labeled transitions measured by the NLP algorithm (see Additional file 2) were discarded by the operator but read by the BHI algorithm. This can be the reason for which these proteins had bad results with the BHI algorithm. Thus, it would be interesting to compare the two algorithms only on spikes selected by the operator.

The BHI algorithm (but not the NLP) allows for the variability stemming from the SRM pre-analytic step (precisely, the digestion step) by using QC samples to estimate the daily digestion yield. This may reduce the variability due to the two-day measurement process, but not systematically; in the presence of endogenous proteins with fragments, the ratio of transitions (native to labeled transition) may be altered, which leads to incorrect estimations of the digestion yields. Other strategies to monitor the digestion step should then be used to estimate correctly the digestion yield, such as the addition of Protein Standard Absolute Quantification, PSAQ [25]. Spiking isotopically labelled proteins has the advantage that incomplete or unspecific digestion does not corrupt the results; this corruption may occur when labelled AQUA internal standards are used.

In each algorithm, when a linear relationship was not clearly observed (< 0.70 correlation coefficient between the theoretical concentration and the measured protein concentration), it was assumed that the protein concentration was below the analytical limit of detection. Actually, the SRM sensitivity depends not only on the protein amount but also on the tryptic peptide sequence and the matrix effect [26].

## Conclusion

After the generic experimental design imagined by Adonna et al. [7], an extension of this design and a variance decomposition via adjusted sums of squares in case of unbalanced data are now available to evaluate the technical variability of protein concentration by SRM measurements and ensure an initial comparison of protein quantification algorithms.

## Additional file

Additional file 1: The full details for sample preparation and SRM analysis. (DOCX 16 kb)

Additional file 2: Number of transitions and peptides per protein and the percent of missing and zero values among protein concentration measurements. (DOCX 18 kb)

Additional file 3: Relationship between theoretical and BHI-quantified protein concentrations. (TIFF 2929 kb)

Additional file 4: Relationship between theoretical and NLP-quantified protein concentrations. (TIFF 2929 kb)

Additional file 5: Scatter plot showing the parts of dilution variance, technical variance, and lack of fit with Model 1S. (TIFF 1318 kb)

## Abbreviations

AQUA: Absolute QUAntification
BHI: Bayesian hierarchical algorithm
Calgi: Calgizzarin or S100 A11
Calmo: Calmodulin
Calret: Calreticulin
Def.A6: Defensin α6
ELISA: Enzyme-Linked immunosorbent assay
I-FABP: Intestinal-fatty acid binding protein
L-FABP: Liver-fatty acid binding protein
MRM: Multiple reaction monitoring
MS: Mass spectrometry
NLP: Non-linear processing
PDI: Protein disulfide-isomerase
Peroxi-5: Peroxiredoxin-5
PSAQ: Protein standard absolute quantification
QC: Quality controls
S100A14: S100 calcium-binding protein A14
SRM: Selected reaction monitoring
TPI: Triophosphate isomerase

## References

[1] Rifai N, Gillette MA, Carr SA (2006). Protein biomarker discovery and validation: the long and uncertain path to clinical utility. Nat Biotechnol.

[2] Lemoine J, Fortin T, Salvador A, Jaffuel A, Charrier JP, Choquet-Kastylevsky G (2012). The current status of clinical proteomics and the use of MRM and MRM^3^ for biomarker validation. Expert Rev Mol Diagn.

[3] Brun V, Masselon C, Garin J, Dupuis A (2009). Isotope dilution strategies for absolute quantitative proteomics. J Proteome.

[4] Eckel-Passow JE, Oberg AL, Therneau TM, Bergen HR (2009). An insight into high-resolution mass-spectrometry data. Biostatistics.

[5] Lange V, Picotti P, Domon B, Aebersold R (2008). Selected reaction monitoring for quantitative proteomics: a tutorial. Mol Syst Biol.

[6] Xia JQ, Sedransk N, Feng X (2011). Variance component analysis of a multi-site study for the reproducibility of multiple reaction monitoring measurements of peptides in human plasma. PLoS One.

[7] Addona TA, Abbatiello SE, Schilling B, Skates SJ, Mani DR, Bunk DM (2009). Multi-site assessment of the precision and reproducibility of multiple reaction monitoring-based measurements of proteins in plasma. Nat Biotechnol.

[8] Grangeat P, Szacherski P, Gerfault L, Giovannelli J-F (2011). Bayesian hierarchical reconstruction of protein profiles including a digestion model. 59th ASMS conference on mass spectrometry, Denver, Colorado, USA.

[9] Gerfault L, Szacherski P, Giovannelli J-F, Charrier J-P, Mahé P, Grangeat P (2012). A hierarchical SRM acquisition chain model for improved protein quantification in serum samples.

[10] Szacherski P (2012). Reconstruction de profils protéiques pour la recherche de biomarqueurs, Ph. D. thesis, Université de Bordeaux 1.

[11] Szacherski P, Gerfault L, Giovannelli J-F, Giremus A, Mahé P, Fortin T (2013). MRM protein quantification and serum sample classification, 61^st^ ASMS conference on mass spectrometry and allied topics, Minneapolis, Minnesota, USA.

[12] Grangeat P, Giovannelli J-F, Roy P, Picaud V, Truntzer C, Lemoine J (2013). Convergence entre l’analyse biostatistique et les méthodes d’inversion hiérarchique bayésienne pour la recherche et la validation de biomarqueurs par spectrométrie de masse.

[13] Gerfault L, Szacherski P, Giovannelli J-F, Giremus A, Mahé P, Fortin T, et al. Assessing MRM protein quantification and serum sample classification performances of a Bayesian Hierarchical Inversion method on a colorectal cancer cohort. Saint-Malo: EuPA 2013 Scientific Meeting; 2013.

[14] Gerfault L, Klich A, Mercier C, Roy P, Giovannelli J-F, Giremus A (2014). Statistical analysis of Bayesian hierarchical inversion for MRM protein quantification and QDA serum sample classification. 62^nd^ ASMS conference on mass spectrometry and allied topics, Baltimore, Maryland, USA.

[15] Szacherski P, Giovannelli JF, Gerfault L, Mahé P, Charrier P, Giremus A (2014). Classification of proteomic MS data as Bayesian solution of an inverse problem. Access, IEEE.

[16] Grossmann J, Roschitzki B, Panse C, Fortes C, Barkow-Oesterreicher S, Rutishauser D (2010). Implementation and evaluation of relative and absolute quantification in shotgun proteomics with label-free methods. J Proteome.

[17] Irizarry RA, Hobbs B, Collin F, Beazer-Barclay YD, Antonellis KJ, Scherf U (2003). Exploration, normalization, and summaries of high density oligonucleotide array probe level data. Biostatistics.

[18] Weisberg S (2005). Applied linear regression.

[19] Chang CY, Picotti P, Hüttenhain R, Heinzelmann-Schwarz V, Jovanovic M, Aebersold R (2012). Protein significance analysis in selected reaction monitoring (SRM) measurements. Mol Cell Proteomics.

[20] Hector A, Von Felten S, Schmid B (2010). Analysis of variance with unbalanced data: an update for ecology and evolution. J Anim Ecol.

[21] Cramer EM, Appelbaum MI (1980). Nonorthogonal. Analysis of variance once again. Psychol Bull.

[22] Langsrud Ø (2003). ANOVA for unbalanced data: use type II instead of type III sums of squares. Stat Comput.

[23] Nelder JA, Lane PW (1995). The computer analysis of factorial experiments: in memoriam - frank yates. Am Stat.

[24] Nelder J (1994). The statistics of linear models: back to basics. Stat Comput.

[25] Beynon RJ, Doherty MK, Pratt JM, Gaskell SJ (2005). Multiplexed absolute quantification in proteomics using artificial QCAT proteins of concatenated signature peptides. Nat Methods.

[26] Dupin M, Fortin T, Larue-Triolet A, Surault I, Beaulieu C, Gouel-Chéron A (2016). Impact of serum and plasma matrices on the titration of human inflammatory biomarkers using analytically validated SRM assays. J Proteome Res.

