# Supplementary material for: Variance component analysis to assess protein quantification in biomarker validation: application to selected reaction monitoring-mass spectrometry
Source: BMC Bioinformatics. 2018 Mar 1;19:73. doi: 10.1186/s12859-018-2075-8 (PMC5831836; doi:10.1186/s12859-018-2075-8)
Supplement: Supplementary file 1 — The full details for sample preparation and SRM analysis. (DOCX 16 kb) [file 12859_2018_2075_MOESM1_ESM.docx]

**Additional file 1 : details of sample preparation and SRM analysis**

The digestion used trypsin to cut proteins, mainly at the [carboxyl](http://en.wikipedia.org/wiki/Carboxyl) side of [amino acids](http://en.wikipedia.org/wiki/Amino_acids) R (arginine) and K ([lysine](http://en.wikipedia.org/wiki/Lysine)), except when either is followed by P ([proline](http://en.wikipedia.org/wiki/Proline)) to yield peptides with well-determined amino acid sequences. Basically, 10 µL of heavy-labeled internal peptide standard was added to 100 µL of each serum serial dilution. These samples were denatured using 400 µL of 8 M urea and reduced using 55 µL of 150 mM dithiotreitol during 40 min at 60° C. Proteins were further alkylated to block cysteine thiols using 170 µL of 150 mM iodoacetamide during 40 min in the dark at room temperature. Then, 200 µg of trypsin (Sigma, Lyon, France), diluted in 3 mL of 50 mM ammonium bicarbonate, pH 8.0, was added to the samples and the digestion performed at 37° C during 4 h. Reduction, alkylation, and digestion steps were repeated once and the tryptic digestion was stopped after an overnight digestion by acidifying the samples with 25 µL formic acid. The samples were further fractionated by solid-phase extraction using MCX Oasis cartridges (Waters, Milford, USA) to desalt, reduce sample complexity, and concentrate the peptides. After each sample was injected into the chromatographic cartridge with the mixed cation exchange resin, the unbound compounds were removed by pure methanol and 0.2 M acetic acid, pH 3.0, washes. A 1/1 methanol, 0.2 M sodium acetate, pH 5.5 solution (v/v) was then added to each sample for peptide elution. Elution of various peptide fractions are possible using various buffers, but in this work, only the fraction with pH 5.5 was analyzed by SRM because it contains the majority of the peptides of interest. Finally, the samples were concentrated using a SpeedVac concentrator (Thermo Scientific, Courtaboeuf, France) to approximately 100 µL and further diluted to exactly 250 µL in 94.5/5/0.5 water/acetonitrile/formic acid (v/v).

The SRM analysis was performed using a Nexera HPLC instrument with a binary pump and an autosampler (Shimadzu, Kyoto, Japan) hyphenated to a hybrid triple quadripole/linear ion trap MS (QTRAP 5500, AB Sciex, Foster City, CA).

During SRM, each peptide ion was selected in the first Quadripole (Q1), fragmented in the second quadripole (Q2) and specific fragments were successively selected in the third quadripole (Q3) and detected. This double mass selection, in Q1 and Q3, defines a transition; at the most, three transitions were monitored per peptide.

Instrument control and data acquisition and processing were performed using Analyst 1.5.1 software. The chromatographic separation of 100 µL of the peptide solutions was carried out on a Kinetex C18 column (100 * 2.1 mm, particle size: 2.6 µm, porosity: 130 Å) from Phenomenex (Torrance, CA). Elution was performed at a flow rate of 300 µL/min with water containing 0.1% (v/v) formic acid as solvent A and acetonitrile containing 0.1% (v/v) formic acid as solvent B. A 3-min isocratic step at 5% solvent B was followed by a 25-min linear gradient from 5% to 50% solvent B and a 3-min isocratic step at 100% solvent B. Two columns were used alternately, one running a sample while the other is washed and equilibrated. The mass spectrometer was initially tuned and calibrated using polypropylene glycol, reserpine, and the Agilent Tuning Mix (all AB Sciex) according to the manufacturer’s instructions. The first quadripole (Q1) resolution was adjusted to 0.7 ± 0.1 atomic mass unit full width at half maximum (amu-fwhm), referred to as unit resolution. A step of ion fragmentation was carried out into the second quadripole (Q2) for each peptide. The third quadripole (Q3) was also set to unit resolution. MS analysis was carried out in positive ionization mode using an ion spray voltage of 5500 V and 3-min scheduled SRM windows. The nebulizer and the curtain gas flows were set at 45 psi using nitrogen. The QTRAP 5500 Turbo V ion source was operated at 550°C with the auxiliary gas flow (nitrogen) set at 40 psi.
