# Supplementary material for: Variance component analysis to assess protein quantification in biomarker validation: application to selected reaction monitoring-mass spectrometry
Source: BMC Bioinformatics. 2018 Mar 1;19:73. doi: 10.1186/s12859-018-2075-8 (PMC5831836; doi:10.1186/s12859-018-2075-8)
Supplement: Supplementary file 2 — Number of transitions and peptides per protein and the percent of missing and zero values among protein concentration measurements. (DOCX 18 kb) [file 12859_2018_2075_MOESM2_ESM.docx]

**Additional file 2: Table 1** – Number of transitions and peptides per protein and the percent of missing and zero values among transition peak measurements by the NLP algorithm per protein and per peptide.

| Protein | Peptide | Number of transition | Percent of missing values  of the labeled transition | | Percent of zero values of the native transition | |
| --- | --- | --- | --- | --- | --- | --- |
| LFABP |  |  | 27 |  | 41.7 |  |
|  | AIGLPEELIQK | 3 | 55.1 |  | 65.5 |  |
|  | GVSEIVQNGK | 2 | 24.6 |  | 25.8 |  |
|  | TVVQLEGDNK | 3 | 0.4 |  | 29.6 |  |
| Villin |  |  | 16 |  | 61 |  |
|  | AAVPDTVVEPALK | 2 | 8.7 |  | 47.3 |  |
|  | DPETPIIVVK | 3 | 8.7 |  | 58 |  |
|  | EVQGNESEAFR | 3 | 25.9 |  | 50.8 |  |
| 14.3.3 sigma | YLAEVATGDDK | 3 | 13.3 |  | 26.2 |  |
| Calgi |  |  | 0 |  | 16 |  |
|  | DPGVLDR | 3 | 0 |  | 5.17 |  |
|  | ISSPTETER | 3 | 0 |  | 27 |  |
|  |  |  |  |  |  |  |
| Def.A6 | AYEADAQEQR | 3 | 0.8 |  | 4.2 |  |
|  |  |  |  |  |  |  |
| Calmo | DTDSEEEIR | 3 | 0 |  | 3.4 |  |
|  |  |  |  |  |  |  |
| IFABP | LTITQEGNK | 3 | 1.6 |  | 22 |  |
|  |  |  |  |  |  |  |
| Peroxi-5 | LLADPTGAFGK | 3 | 27 |  | 30 |  |
| S100A14 |  |  | 36 |  | 49.7 |  |
|  | ETLTPSELR | 3 | 46.2 |  | 50.6 |  |
|  | IANLGSCNDSK | 2 | 20 |  | 49.5 |  |
