# Supplementary figures and images for: Variance component analysis to assess protein quantification in biomarker validation: application to selected reaction monitoring-mass spectrometry
Source: BMC Bioinformatics. 2018 Mar 1;19:73. doi: 10.1186/s12859-018-2075-8 (PMC5831836; doi:10.1186/s12859-018-2075-8)

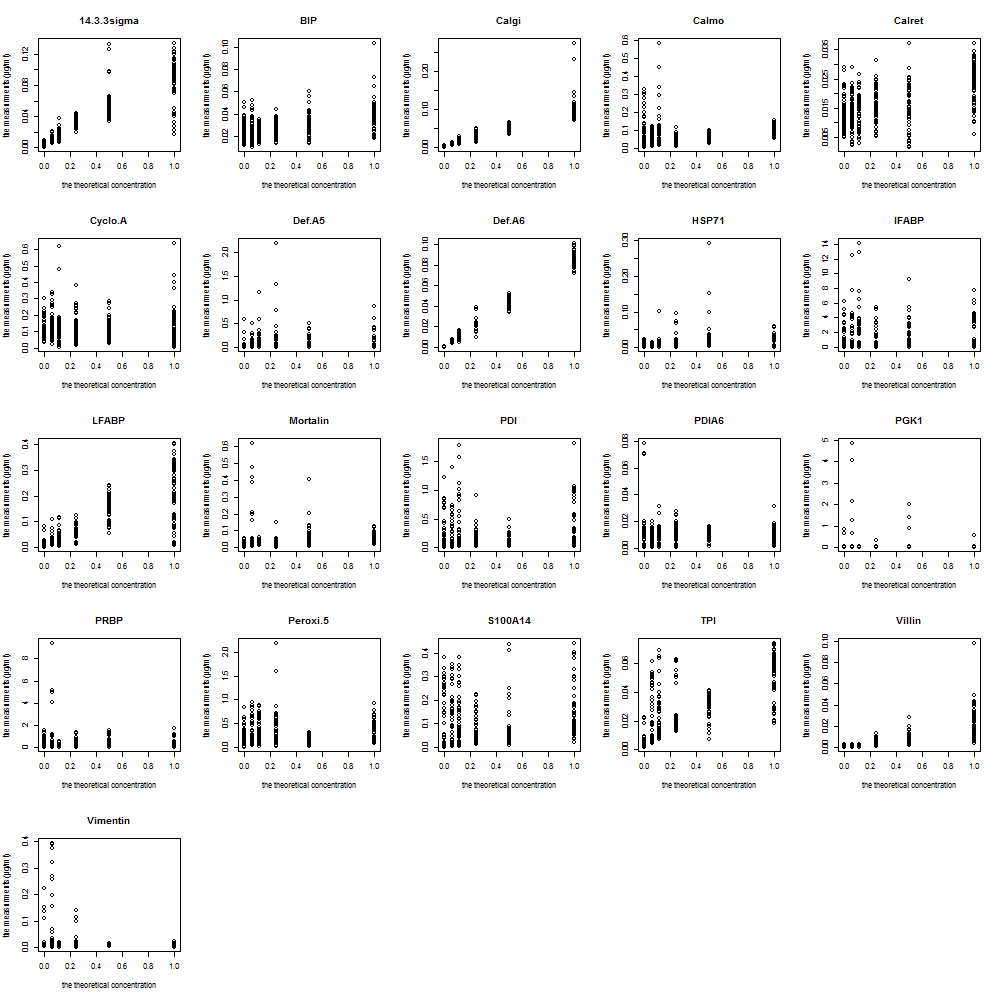

Supplement: Supplementary file 3 — Relationship between theoretical and BHI-quantified protein concentrations. (TIFF 2929 kb) [file 12859_2018_2075_MOESM3_ESM.tiff]

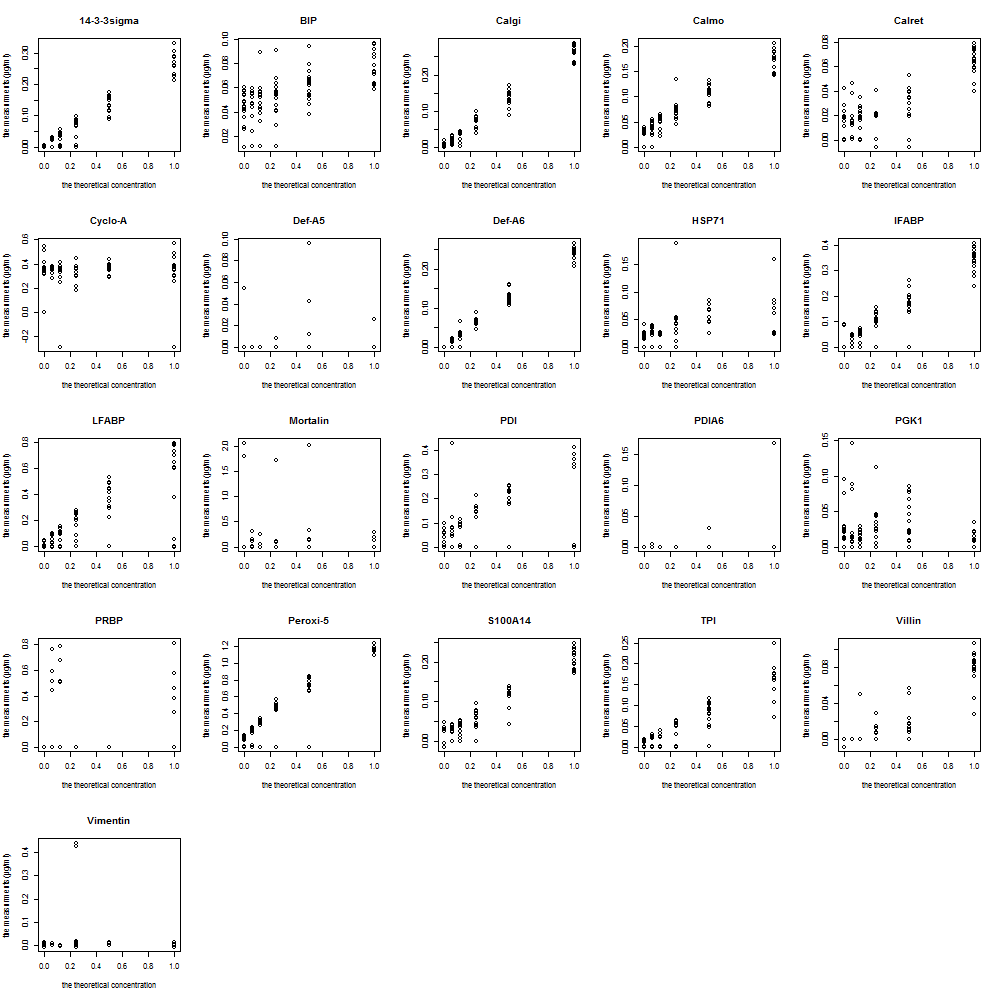

Supplement: Supplementary file 4 — Relationship between theoretical and NLP-quantified protein concentrations. (TIFF 2929 kb) [file 12859_2018_2075_MOESM4_ESM.tiff]

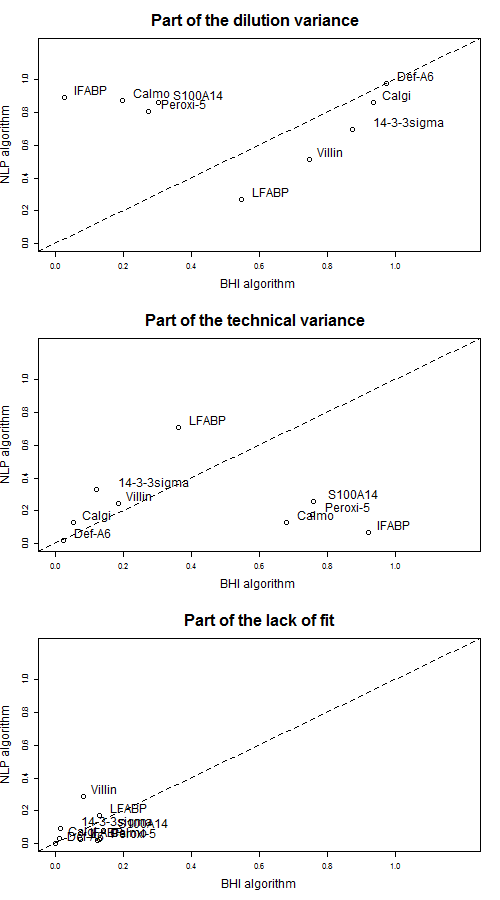

Supplement: Supplementary file 5 — Scatter plot showing the parts of dilution variance, technical variance, and lack of fit with Model 1S. (TIFF 1318 kb) [file 12859_2018_2075_MOESM5_ESM.tiff]
